# Not always ‘squame’: The rare entity of follicular dendritic cell sarcoma of the tonsil presenting with cervical nodal metastases

**DOI:** 10.4102/sajr.v24i1.1978

**Published:** 2020-12-17

**Authors:** Jaanri Brugman, Gerard de Bruyn, Komeela Naidoo, Marc Merven, Johan Opperman, Leon Janse van Rensburg

**Affiliations:** 1 Division of Radiodiagnosis, Department of Medical Imaging and Clinical Oncology, Faculty of Medicine and Health Sciences, Stellenbosch University, Cape Town, South Africa; 2 Division of Otorhinolaryngology, Faculty of Medicine and Health Sciences, Stellenbosch University, Cape Town, South Africa; 3 Division of Radiation Oncology, Department of Medical Imaging and Clinical Oncology, Faculty of Medicine and Health Sciences, Stellenbosch University, Cape Town, South Africa; 4 Division of Anatomical Pathology, Faculty of Medicine and Health Sciences, Stellenbosch University, Cape Town, South Africa

**Keywords:** follicular dendritic cell sarcoma, magnetic resonance, tonsil, nodal metastasis, squamous cell carcinoma, head and neck neoplasm, oropharynx

## Abstract

Although squamous cell carcinoma accounts for the overwhelming majority of head and neck malignant neoplasms, extranodal follicular dendritic cell sarcoma (FDCS) of the pharyngeal region can have a similar clinical presentation. The histopathological features of this rare entity have been described and emphasised in the literature. We present the case of a 65-year-old male patient with FDCS of the tonsil to illustrate the radiologic findings of FDCS and also highlight this infrequent but salient differential diagnosis for adult head and neck neoplasia.

## Introduction

Follicular dendritic cell sarcoma (FDCS) is an exceedingly rare malignant neoplasm of follicular dendritic cells, first characterised by Monda et al. in 1986.^1^ Follicular dendritic cells are immune accessory cells widely distributed in both nodal and extranodal lymphoid tissue, presenting antigens to B-lymphocytes.^2^ Thus, uncontrolled proliferation of these cells affects lymph nodes, primarily of the mediastinum and cervical and axillary regions.^3^ When primary extranodal disease occurs, the pharyngeal region is one of the preferred sites, in particular the tonsils.^4^

## Case presentation

A 65-year-old man presented with a 5-month history of an enlarged and growing left-sided neck mass. The patient reported no weight loss, odynodysphagia, dysphonia or otalgia. On examination of the neck, it was observed that the mass measured 10 × 10 cm, was non-tender to palpation and was of a mixed consistency (both cystic and solid). Furthermore, the mass appeared to be mobile but felt attached to the inferior aspect of the ipsilateral parotid gland. Oropharyngeal examination revealed a small (< 1 cm) submucosal sessile polyp on the left tonsil. The rest of the clinical examination was normal.

Biopsy of the left tonsil and cell block obtained from fine-needle aspiration of the left-sided neck mass revealed FDCS using immunohistochemical staining.

**FIGURE 1:**
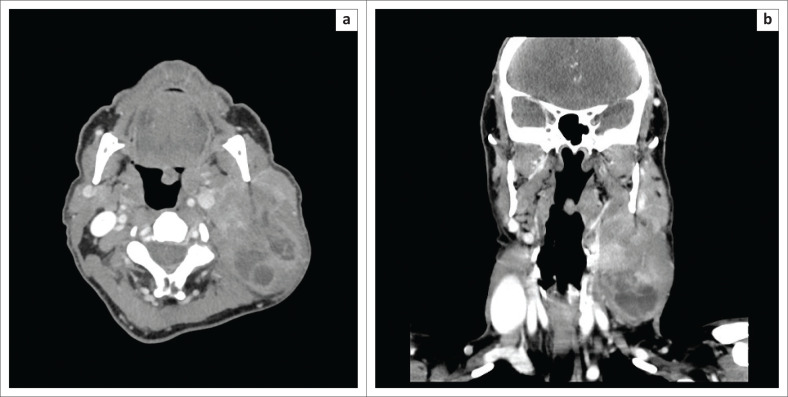
(a) Axial contrasted computed tomography (CT) scan demonstrates a small ovoid iso-attenuating mass in the left tonsil. The large mixed solid–cystic nodal-mass conglomerate in the left side of the neck exerts mass effect on the left carotid space and displaces it medially. (b) Coronal contrasted CT scan demonstrates the cranio-caudal extent of the nodal-mass conglomerate in the left side of the neck. The cranial aspect is solid and enhancing, and the caudal portion is cystic and multiseptated.

**FIGURE 2:**
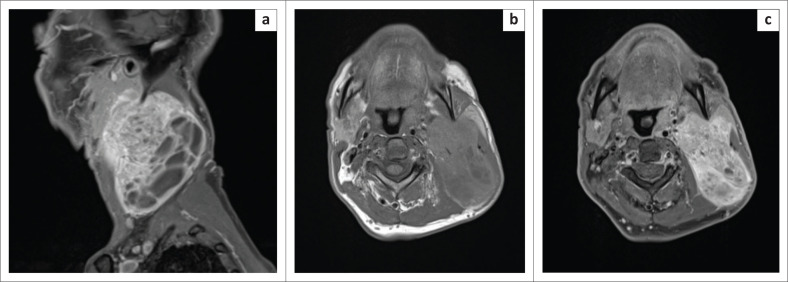
(a) Sagittal T1-weighted, post-gadolinium, fat-saturated magnetic resonance (MR) image shows the mixed solid–cystic nature of the cervical nodal-mass with enhancement of the cranial solid portion. Superficial parotid gland infiltration was suspected based on MR imaging findings. Axial T1-weighted MR images with (b) gadolinium and (c) fat suppression demonstrating heterogenous enhancement of the solid cranial portion of the cervical nodal-mass conglomerate. The left carotid arteries and internal jugular vein were not infiltrated.

Computed tomography (CT) of the neck and chest with intravenous contrast administration was carried out, followed by magnetic resonance (MR) imaging of the neck with intravenous gadolinium administration to further delineate the extent of the tumour and its relation to adjacent structures, in particular the parotid gland and the cervical vasculature. The neck CT demonstrated an ovoid iso-attenuating mass in the left tonsil, measuring 16 mm × 12 mm × 24 mm in diameter (anteroposterior × transverse × craniocaudal, Figure 1a). In addition, a large, mixed solid–cystic nodal-mass conglomerate was present in the left side of the neck, with a solid and enhancing component at its cranial aspect and, inferiorly, a multiseptated cystic component (Figure 1b). The solid cranial component of the cervical nodal-mass demonstrated diffusion restriction with low apparent diffusion coefficient values. On MR imaging, left parotid gland invasion by the cervical nodal conglomerate was strongly suspected (Figure 2a). The carotid artery and internal jugular vein were not infiltrated. The tonsillar mass demonstrated T1-weighted iso- to hyperintense signal to cervical muscles, T2-weighted and fluid attenuated inversion recovery (FLAIR) hyperintense signal to muscle and minimal post-contrast enhancement (Figure 2b and 2c). No distant metastasis was present in the chest or upper abdomen. A neck ultrasound was not carried out prior to cross-sectional imaging.

## Surgical management

**FIGURE 3:**
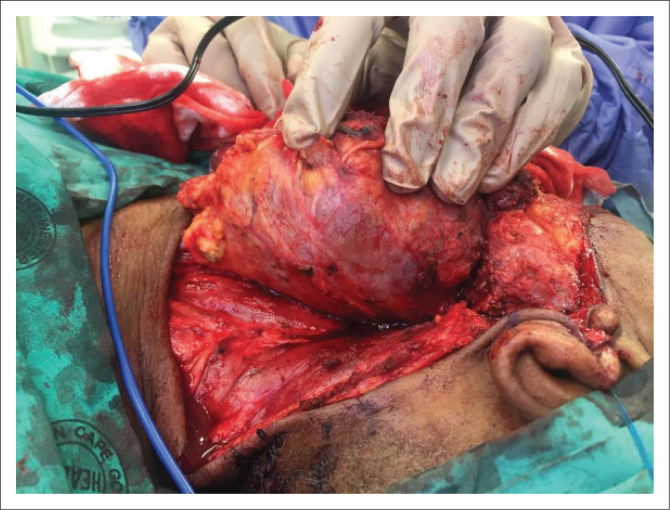
Intra-operative image of the left cervical nodal-mass conglomerate. It was dissected off the left common carotid artery, with ligation of the left internal jugular vein.

After discussion at the head and neck and oncology multidisciplinary meetings, the patient was staged with having a clinical T2N1M0 sarcoma (American Joint Committee on Cancer tumor-node-metastasis [AJCC TNM] 8th edition). The patient underwent a left-sided, modified radical neck dissection with a superficial partial parotidectomy (‘en block’) and bilateral tonsillectomy (Figure 3). The post-operative course was uncomplicated, and the patient was discharged on Day 5 post surgery.

## Histopathology

Gross macroscopic examination showed a solid and firm, well-circumscribed tumour involving the left tonsil, measuring approximately 2 cm. Left neck level II and III nodal metastases were present with most of the nodes in level III involved by tumour comprising solid and cystic spaces filled with gelatinous material. The tumour abutted the parotid gland capsule; however, frank invasion was not observed macroscopically.

**FIGURE 4:**
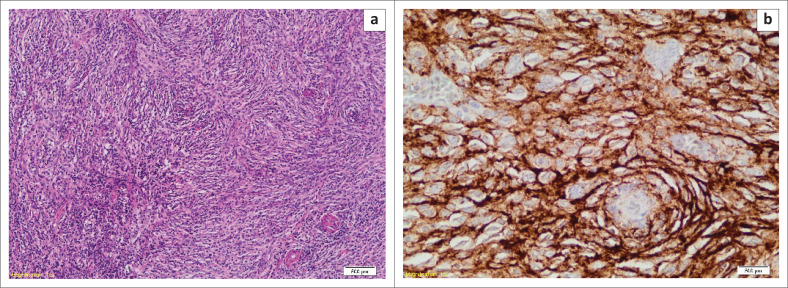
Follicular dendritic cell sarcoma of the left tonsil: (a) The tumour appears very cellular, displaying a typical storiform and whorled growth pattern (haematoxylin and eosin, 100×); (b) By immunohistochemistry, the tumour shows diffuse membranous positivity with follicular dendritic cell markers D2-40 (haematoxylin and eosin, 400×), as well as CD21, CD23, CD35 (not shown).

Microscopic examination revealed a malignant neoplasm partially involving the left tonsil, with intact overlying surface epithelium. The tumour comprised spindle-to-ovoid cells arranged in fascicles, in whorled and storiform patterns, demonstrating a pushing invasive front (Figure 4a). Occasional mitosis was noted (1–2 per 10 hpf), but necrosis was absent. The left cervical lymph nodes showed a conglomerate of nodes involved by tumour, with extracapsular tumour spread and extension into the surrounding muscle, fat and adjacent parotid gland. The tumour cells were positive for follicular dendritic cell markers such as CD21, CD23, CD35 and D2-40 (podoplanin) but negative for cytokeratin markers (Figure 4b). Based on the morphology and immunohistochemistry, the diagnosis of an extranodal FDCS was rendered.

Following the histological review, the patient’s pathological staging was T2N1. The role of adjuvant radiotherapy in sarcoma of the head and neck is controversial, with indications being extrapolated from the treatment guidelines of extremity sarcoma. In view of the large nodal disease with extracapsular extension, the multidisciplinary team’s decision was to offer adjuvant radiotherapy. The patient received 66 Gy in 2 Gy/# to the tumour bed and left level IB-V. This was delivered via volumetric modulated radiotherapy. Radiotherapy was tolerated well with minimal side effects, and during the last assessment, the patient was still in remission.

## Discussion

Duan et al.^4^ identified a total of 41 cases of extranodal FDCS in the English literature, 51% of which were tonsillar in origin. Other primary extranodal sites of disease include the spleen, gastro-intestinal tract, liver, soft tissue, skin, lung and breast. Follicular dendritic cell sarcoma has a slight female predominance (1.2:1) and a wide patient age range for disease presentation, although it most often presents in the fifth decade of life.^2^ The World Health Organisation classification of haematopoietic and lymphoid neoplasms includes FDCS in the group of histiocytic and dendritic cell neoplasms.^5^

Because of the rarity of dendritic cell sarcoma (DCS), its clinical behaviour and optimal management has not been consolidated. De Pas et al.^3^ reviewed 189 cases of DCS (including both FDCS and interdigitating DCS) and found that DCS was most often localised at diagnosis. In our case report, the patient’s tumour had metastasized to regional lymph nodes, with clinical and pathological features of extranodal invasion, suggesting a more aggressive biological nature of the tumour.

Anecdotal evidence suggests that abdominal tumours or those measuring more than 5 cm relate to an unfavourable prognosis. In addition, specific histopathological features, such as a high mitotic count and the presence of necrosis, have been studied in other soft-tissue sarcomas and proven their value in prognostication.^3^ Of note, our patient’s tumour demonstrated a low mitotic rate and absence of necrosis, despite its large nodal metastases with extranodal invasion.

The histological features of FDCS have been emphasised more often than the imaging findings. As of yet, characteristic findings of FDCS on 18F-fluoro-deoxyglucose positron emission tomography (FDG-PET) have not been established, as a variable radiotracer uptake has been found, presumably as a result of the diverse histological grade features demonstrated in these tumours.^6^ The use of 18F-FDG-PET in surveillance imaging, recurrence detection and response to therapy has been highlighted in case report studies on parotid gland and cervical node FDCS.^7,8^

## Conclusion

Due to its rarity, extranodal FDCS of the pharyngeal region is often not considered as part of the differential diagnosis in adult head and neck neoplasia.^4^ Our case not only highlights the clinical and histopathological findings of FDCS but also illustrates the imaging findings of the condition, in particular, the use of MR imaging, of which there is a scarcity in the literature.
