# Corrigendum: Not always ‘squame’: The rare entity of follicular dendritic cell sarcoma of the tonsil presenting with cervical nodal metastases

**DOI:** 10.4102/sajr.v25i1.2268

**Published:** 2021-12-01

**Authors:** Jaanri Brugman, Gerard de Bruyn, Komeela Naidoo, Marc Merven, Johan Opperman, Leon Janse van Rensburg

**Affiliations:** 1 Division of Radiodiagnosis, Department of Medical Imaging and Clinical Oncology, Faculty of Medicine and Health Sciences, Stellenbosch University, Cape Town, South Africa; 2 Division of Otorhinolaryngology, Faculty of Medicine and Health Sciences, Stellenbosch University, Cape Town, South Africa; 3 Division of Radiation Oncology, Department of Medical Imaging and Clinical Oncology, Faculty of Medicine and Health Sciences, Stellenbosch University, Cape Town, South Africa; 4 Division of Anatomical Pathology, Faculty of Medicine and Health Sciences, Stellenbosch University, Cape Town, South Africa

In the version of the article initially published, Brugman J, De Bruyn G, Naidoo K, et al. Not always ‘squame’: The rare entity of follicular dendritic cell sarcoma of the tonsil presenting with cervical nodal metastases. S Afr J Rad. 2020;24(1), a1978. https://doi.org/10.4102/sajr.v24i1.1978, the ORCID of the last author was given incorrectly. The correct ORCID should be https://orcid.org/0000-0003-1549-6785 instead of https://orcid.org/0000-0003-1317-2699 in the ‘Authors’ section.

This correction does not alter the study’s findings of significance or overall interpretation of the study’s results. The authors apologise for any inconvenience caused.

